# Trust and Privacy Concerns Among Cancer Survivors Who Did Not Visit a Research Website Offering Free Genetic Counseling Services for Families: Survey Study

**DOI:** 10.2196/64228

**Published:** 2025-05-06

**Authors:** James A Shepperd, Colleen M McBride, Weihua An, Jingsong Zhao, Rebecca D Pentz, Cam Escoffery, Kevin Ward, Yue Guan

**Affiliations:** 1 Department of Psychology University of Florida Gainesville, FL United States; 2 Department of Behavioral, Social and Health Education Sciences Rollins School of Public Health Emory University Atlanta, GA United States; 3 Department of Sociology and Department of Quantitative Theory and Methods Emory University Atlanta, GA United States; 4 Winship Cancer Institute School of Medicine Emory University Atlanta, GA United States; 5 Department of Epidemiology Rollins School of Public Health Emory University Atlanta, GA United States

**Keywords:** internet trust, internet privacy, hereditary cancers, patients and relatives outreach, social marketing

## Abstract

**Background:**

Digital health tools, such as websites, now proliferate to assist individuals in managing their health. With user input, we developed the Your Family Connects (YFC) website to promote access to genetic services for survivors of ovarian cancer and their relatives*.* Although we estimated that half or more would access the website, only 18% of invited survivors did so. We assessed the extent to which perceived relevance of the information provided, trust, and privacy concerns influenced decisions not to access the website.

**Objective:**

We designed a theory-based cross-sectional survey to explore the following questions: (1) To what extent did nonresponders endorse privacy concerns? (2) Were privacy concerns associated with recall of receiving the website invitation, time since diagnosis, age, and race? (3) Could we identify profiles of nonresponders that would guide the development of future interventions to encourage engagement in health websites for families affected by inherited cancers?

**Methods:**

A sample of survivors who were eligible to access the website yet did not respond to the study invitation was identified by linking study IDs to the Georgia Cancer Registry information. The survey was brief and contained 27 items, including recall of the invitation, interest in ovarian cancer information, benefits of using health websites, trust in health websites, and trust in university-based health research. We conducted factor analyses, regression analyses, ANOVA, correlation analyses, and logistic regression to address research questions.

**Results:**

Of the 650 nonresponders to whom we sent the short survey, 368 (56.3%) responded and provided sufficient data for analysis. The mean response of 2.57 on the trust scale was significantly below the scale midpoint of 3 (*t*_360_=11.78, *P*<.001), suggesting that survivors who did not log on were on average distrustful of health websites. Belonging to a racial or ethnic minority group was associated with being more trusting and less skeptical about health websites. Just 196 (30.1%) nonresponders recalled the invitation to visit the website. Logistic regression analysis indicated that age was the only significant predictor of recall. Testing a model with age, racial or ethnic minority status, and the 6 privacy concerns correctly classified 58.8% of nonresponders, a rate of successful classification that was not appreciably better than a logistic regression analysis that included only age as a predictor.

**Conclusions:**

The nonresponders in the present study—particularly the White nonresponders—were skeptical of website platforms regardless of whether they recalled receiving a website invitation or not. Social marketing approaches geared toward building trust in web platforms by building a relationship with an information consumer and in collaboration with trusted organizations warrant further investigation.

**Trial Registration:**

ClinicalTrials.gov NCT04927013; https://clinicaltrials.gov/study/NCT04927013

## Introduction

### Background

Digital health tools such as websites are intended to assist people in managing their health by providing information and access to services. These tools have become a ubiquitous means to disseminate health promotion services [1]. Moreover, a review of health websites evaluated in randomized control trials reveals that people consistently showed improvement in a variety of health-related behaviors when they used interactive and tailored websites compared with generic websites [2]. But how successful are these digital health tools at disseminating health information?

We conducted an intervention in which we invited by mail women diagnosed with ovarian cancer and identified through a state registry to visit a website designed to expand the reach of genetic services to a population-based sample of survivors of ovarian cancer and their first- and second-degree relatives. We found that relatively few survivors (18%, 147 of 969 in the treatment condition; 202 of 969 in the standard care condition) used the study access codes we provided to view the website information [3]. Our findings are consistent with several other interventions to disseminate inherited risk information to at-risk families [4-6].

Our intervention study revealed that longer time since diagnosis, older age, and status as a racial and ethnic were negatively associated with assessing the website [3]. Consistently, a 2023 review found younger, newly diagnosed cancer patients more likely to seek digital information, though ethnicity findings were inconsistent [7].

### Privacy Concerns and Use of Health Websites

Researchers have noted that privacy preferences—the ability of an individual to control when, to what extent, and how information about the self is communicated to others—can inhibit participation in websites that involve sharing personal information [8]. Theoretical frameworks such as the Privacy Calculus Model posit that willingness to share personal information is a function of the individual’s perception of the ratio of benefits to risks [9]. Benefits include learning about personal and family health risks and receiving valued services. Risks include unwanted disclosure of private information. Diagnosis of a potentially inherited condition may compound the privacy risks because survivors could view information sharing to be a breach of their privacy and that of their blood relatives.

A 2019 survey of 25,000 internet users revealed high trust in the internet (74%) but growing privacy concerns [10]. These concerns are likely due to tracking personal information without consent [11], misinformation [12], and frequent breaches of personal information [13].

In this report, we analyzed data from a cross-sectional survey of women who were identified as ovarian cancer survivors through the Georgia Cancer Registry (GCR). These women were “nonresponders” in the sense that they were sent an invitation to visit a study website that provided information about inherited risk for ovarian cancer for themselves and their at-risk relatives, yet they did not visit the website during the main trial recruitment period. We prioritized privacy concerns for 2 reasons. First, interviews conducted by citizen scientists on our team revealed that privacy represented a central concern to ovarian cancer survivors [14]. The survivors interviewed were uncomfortable with having their interviews recorded and were not placated by our reassurance. Second, despite our recruitment materials emphasizing the protection of privacy and personal information, many survivors in our intervention study voiced strong concerns about privacy.

Our survey was designed to answer three research questions: (1) To what extent did participants endorse privacy concerns? Mistrust of health websites and strong endorsement of privacy concerns would be consistent with the assertion that these considerations dissuaded participation in our parent study. (2) Were privacy concerns associated with the recall of receiving the website invitation, time since diagnosis, age, and race? Finding that the participants who most strongly endorsed privacy concerns also were most likely to recall receiving our study invitation would suggest that our study may have evoked privacy concerns. Conversely, finding that the participants who most endorsed privacy concerns were the participants who did not recall receiving our study invitation would suggest that privacy concerns were not responsible for our low participation rate. Finally, finding no difference in privacy concerns irrespective of whether one recalled or did not recall seeing our study invitation would suggest a general suspicion of websites in our sample of ovarian cancer survivors. (3) Could we identify profiles of nonresponders that would guide the development of future interventions to encourage engagement in health websites for families affected by inherited cancers?

## Methods

### Parent Study

This survey study was a follow-up to the Your Family Connects (YFC) intervention, a randomized trial targeting ovarian cancer survivors [3,15]. The intervention used an interactive website to expand access to genetic services for ovarian cancer survivors and their first- and second-degree relatives. Eligible participants were women identified by the GCR who were aged 18 or older, diagnosed with ovarian, fallopian tube, or peritoneal cancer between January 2005 and December 2017 in Georgia, and confirmed alive according to registry records. Survivors were randomly assigned to either a standard or intervention arm. To access the study website, the standard arm received a registry standard study invitation letter, whereas the intervention arm received a family-centered infographic (see Multimedia Appendix 1).

We designed the website content to show the relevancy of genetic counseling and testing for first- and second-degree relatives of women diagnosed with ovarian cancer. Survivors in both arms were encouraged to reach out and share the website content because the information it contained could guide personalized care and management strategies, such as enhanced breast cancer screening for relatives identified with pathogenic variants in *BRCA* genes. Both arms were informed about website login and content. A fuller description of the YFC intervention and the study materials appears elsewhere [3,15]. Importantly, we did not have access to genetic testing data from the cancer registry and thus could not use this information to screen our study sample. Meta-analysis evidence indicates a 39% referral rate for genetic counseling and a 30% completion rate [16]. Other research finds that genetic counseling and referral rates for ovarian cancer range from 10% to 30% [17]. It is possible that as many as 30% of our sample had already received genetic counseling. However, this number ignores the fact that survivors could have used the website to enumerate relatives for genetic counseling.

In the YFC intervention study, nonresponders differed from responders in 3 ways: nonresponders were diagnosed with ovarian cancer later, were older, and were disproportionately racial or ethnic minorities. They did not differ from responders in cancer stage, marital status, health insurance type, and rurality [3]. We focus here on nonresponders from the YFC intervention study. Individuals invited to participate in research but who choose not to do so receive little to no attention. Understanding these nonresponders’ reactions to intervention outreach strategies is crucial for designing future interventions.

### Sample

Survivors who were eligible to participate in the YFC study [3,15] yet did not visit the study website (ie, nonresponders) were invited to participate in this nonresponder survey regardless of their arm assignment.

Between March and July 2023, we sent recruitment packets randomly to 650 survivors representing three groups: (1) an active refusal group consisting of 230 survivors who had explicitly declined participation in the YFC parent study; (2) a passive refusal group consisting of 220 survivors whose mailing address was passively confirmed by the GCR (eg, a relative answered a call), yet who neither visited the study website nor explicitly declined participation; and (3) a lost group consisting of 200 survivors for whom the GCR could not confirm the mailing address and who did not visit the website.

The first mail-out packet included a cover letter, a paper survey, a postage-paid return envelope, and a US $10 cash incentive. We offered survivors the option to complete the survey digitally in the cover letter. Two weeks after mailing the recruitment packet, 2 master’s-level research assistants (IS and KM) contacted eligible participants by phone if they had not responded via mail or digital platform. They attempted to reach each survivor every 2-3 days, making up to 5 calls. If the contact efforts failed after 5 attempts, the survivors were categorized as inaccessible. The calls aimed to (1) confirm receipt of the survey packet, (2) offer survey completion by phone, (3) remind survivors to complete the survey by mail or digital platform, and (4) answer study-related questions. The recruitment effort was consistent with GCR’s standard outreach practices.

**Figure 1:**
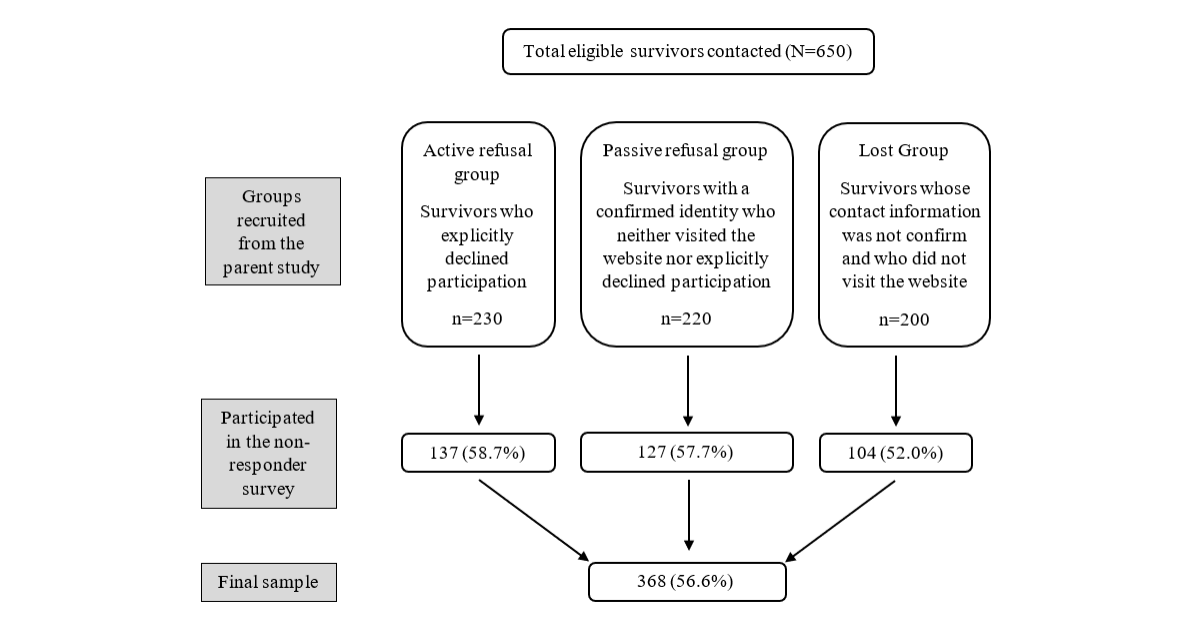
Recruitment flowchart.

Of the 650 nonresponders over the 5-month recruitment period, 105 were lost to follow-up, 87 actively refused to participate, 75 were passive refusals, 5 were deceased after data collection for the parent study (2 from the passive refusal group, 2 from the active refusal group, and 1 from the lost group), 4 were non-English speakers, and 6 lacked internet access. Although the latter 2 reasons meant that the participants were also ineligible for the parent study as well, we were previously unaware of that fact because we could not reach them when recruiting for the parent study. In total, 368 (56.6%) completed the survey with sufficient data for analysis (see Figure 1 for the recruitment flowchart).

Before proceeding to the survey, participants agreed with a consent form stating their rights and explaining that their responses would be confidential and de-identified in the dataset. The average age of our sample was 69.7 (range 30-102, SD 11.8) years, and the average time since diagnosis was 11.9 (range 7-19, SD 3.6) years. The sample was mostly White (n=283, 76.9%) and non-Hispanic (n=257, 69.8%). For our purposes, we classified nonresponders into 2 groups based on self-identification of race and ethnicity: We classified nonresponders as racial or ethnic minorities (n=98) if they self-identified as belonging to a racial or ethnic minority group (ie, as ethnically Hispanic or as non-White in race) and as majority (n=270) if they self-identified as having non-Hispanic ethnicity and White race.

### Data Collection

We mailed nonresponders a brief survey along with a US $10 incentive to complete the survey on paper (and return by mail) or on the web through Qualtrics. The survey comprised 27 items selected to align with perceived benefits (eg, the benefits of using websites for themself and for family) and perceived risks (eg, privacy concerns, skepticism about websites) of the Privacy Calculus Model [9]. We extracted from the GCR and used as predictors only those variables that correlated with logging into the YFC website in our intervention study: time since diagnosis, age, and racial or ethnic status. We assessed vital status by linking the GCR data with the Georgia Department of Public Health Vital Records dataset (in-state deaths) and the National Death Index from the National Center for Health Statistics (out-of-state deaths).

### Ethical Considerations

The study received approval from Emory University (IRB protocol #00000224), and participants were assured that their responses would be kept confidential. All data were deidentified prior to analysis. Participants were compensated with US $10 as an incentive to complete the survey.

### Description of Survey Questions

#### Recall of Invitation

The first item was prefaced by a statement saying that in the past few months, the GCR mailed the survivor a packet of information about a study called “Your Family Connects.” The statement explained that the packet included a US $5 bill and several pages of information about how recipients and their family could access an informational website about inherited risk for ovarian cancer. The item then asked nonresponders if they recalled receiving the envelope and materials (“yes,” “no,” and “unsure”).

#### Perceived Benefits and Risks

**Figure 2:**
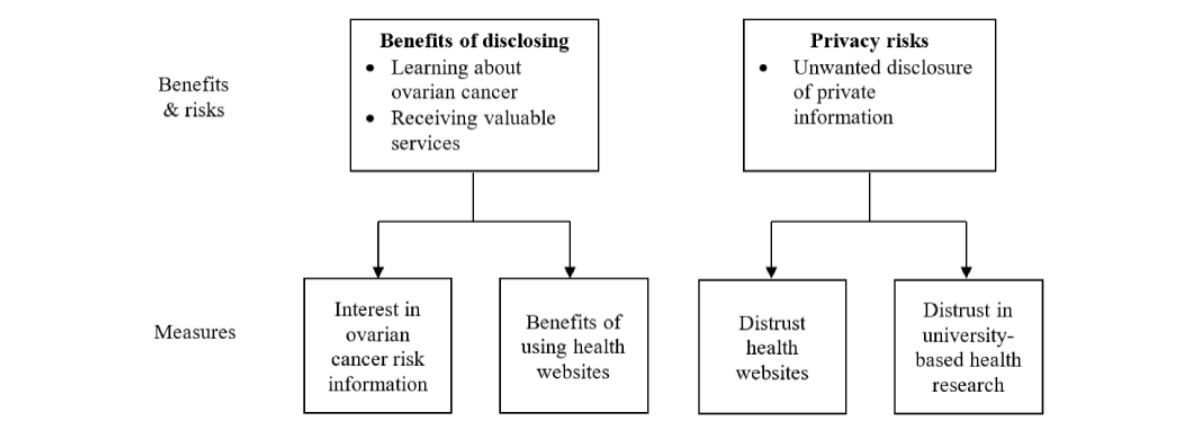
Conceputal model.

The remaining 26 items were adapted from published scales (see Table 1 for the survey instructions and item wording) and were designed to represent 4 concerns. A conceptual model of how we translated the Privacy Calculus Model to the examination of survivors’ concerns with privacy appears in Figure 2. Five of the items were designed to examine perceived interest in ovarian cancer risk information and were modeled after items from the Impression-Relevant Involvement Scales [18]. Six of the items assessed benefits of using health websites and were modeled after a Cochrane review of studies assessing weight loss motivation [19]. Four of the items assessed trust in health websites and were adapted from a measure of attitudes toward science [20]. The remaining 11 items assessed trust in university-based health research and were adapted from items appearing in the guidelines for measuring trust in organizations [21]. Adapting the items entailed one team member (GH) selecting a subset of items from each scale that most closely tapped the construct as pertinent to our research: (1) perceived interest in ovarian cancer risk information, (2) benefits of using health websites, (3) trust in health websites, and (4) trust in university-based health research. The team member then modified the items so that they better reflected our study goal. Next, other team members reviewed the items and offered edits. A back-and-forth discussion of the items proceeded until the team reached an agreement on the final item wording.

The survey items were preceded by instructions asking participants to indicate their agreement with 26 statements about “family resources related to shared risk of ovarian cancer.” All items used a 5-step response format (“1=strongly disagree” to “5=strongly agree”).

**Table 1:** Scale names, items wording, and factor loadings.

Factor name and item wording		Factor loading
**Interest in ovarian cancer information (n=355)**		
	1. My close blood relatives are open to discussing new information about their cancer risk.	.75
	2. Taking part in research to better understand the needs of families affected by ovarian cancer is a high priority for me.	.81
	3. My close blood relatives would benefit from knowing new information about their risk for ovarian and other cancers.	.87
	4. I am not interested in identifying ways for families affected by ovarian cancer to receive genetic counseling^a^.	.58
	5. I could use some help in talking with my family about their risk for ovarian and other cancers.	.49
**Benefits of visiting health websites (n=350)**		
	1. Visiting health websites is an action I can take to stay well-informed about ovarian cancer.	.80
	2. Visiting health websites has little appeal to me at this point in my cancer experience^a^.	.52
	3. Visiting health websites would assure my relatives that I am staying informed about our family’s cancer risks.	.86
	4. Visiting health websites is personally reassuring.	.90
	5. Visiting health websites helps my family stay well-informed about ovarian cancer.	.89
	6. Visiting health websites helps advance ovarian cancer research.	.77
**Website trust (n=355)**		
	1. There is a lot of useful information on health websites.	.85
	2. I trust that most health websites have systems in place to protect my privacy.	.85
**Website skepticism (n=361)**		
	1. People trust health websites a lot more than they should.	.81
	2. A lot of information found on health websites is not based on sound science.	.81
**Trust in health researchers (n=351)**		
	1. University health researchers treat people like me fairly.	.89
	2. University health researchers are concerned about people like me.	.85
	3. University health researchers can be relied on to keep promises.	.92
	4. University health researchers take the opinions of people like me into account when designing studies.	.83
	5. University health researchers do not mislead people like me.	.84
	6. University health researchers keep my personal information confidential.	.77
	7. University health researchers can take advantage of people like me^a^.	.37
**Health research and the greater good (n=356)**		
	1. University health researchers want to contribute to the greater good.	.90
	2. University health researchers can improve health outcomes or care for people like me.	.90
	3. University health researchers generate findings that are generally useful.	.83
	4. University health researchers depend on my participation to be successful.	.73

^a^Reverse-coded items.

### Data Analysis

After reverse-coding 3 items, we conducted 4 separate confirmatory factor analyses (CFA)—1 for each of the 4 scales we examined—to test whether each set of items loaded on a single factor. The CFA was necessary because we used only a subset of items from each scale, and we modified the items to suit our purposes. Because the 26 items were intended to represent 4 distinct constructs, it was not sensible to analyze all 26 items in a single CFA. When necessary, we followed the CFA with an exploratory factor analysis (EFA) and then repeated the CFA on the pattern revealed from the EFA.

To investigate our first research question (the extent to which nonresponders endorsed privacy concerns), we examined the responses to the items assessing trust (and skepticism) in health websites. To investigate our second research question (whether privacy concerns were associated with recall of receiving the website invitation, time since diagnosis, age, and race), we conducted regression analyses in which we entered the demographic variables (age, marginalization, and time since diagnosis) to predict the privacy concerns that potentially influenced their participation decision.

To investigate our third research question (identification of profiles of nonresponders that would guide the development of future websites for families affected by inherited cancers), we initially conducted an ANOVA to explore whether the 3 recall groups differed in age and racial or ethnic minority status. Our point was to determine whether we should examine 3 groups separately or whether we could collapse 2 of the groups. We also correlated all measures to identify variables that were interrelated. Finally, we conducted logistic regression analyses in which we entered our demographic measures and our trait measures of concern as predictors of recall. The identified data and study codebook are available on request from the study principal investigator, YG (yue.guan@emory.edu).

## Results

### Factor Analysis

The results of the factor analyses, including the item wording and factor loadings, appear in Table 1. The factor analyses yielded single factors for the 5 items assessing interest in ovarian cancer information (*χ*^2^_10_=476.6, *P*<.001, α=.68) and for the 6 items assessing the benefits of visiting health websites (*χ*^2^_15_=1194.0, *P*<.001, α=.85). A factor analysis of the 4 items comprising the trust in health websites revealed 2 factors with eigenvalues >1, suggesting that a single factor solution poorly described the data. A follow-up EFA indicated 2 distinct factors from these 4 items (*χ*^2^_1_=121.3, *P*<.001). Guided by the results of EFA, we used CFA to construct a 2-item factor that we labeled "Website trust" (*χ*^2^_1_=76.8, *P*<.001, *r*=.38) and a second 2-item factor that we labeled "Website skepticism" (*χ*^2^_1_=34.7, *P*<.001, *r*=.26).

Finally, a CFA of the 11 items we used to assess trust in university-based health research also revealed 2 factors with eigenvalues >1, again suggesting that a single factor solution poorly described the data. A follow-up EFA also yielded 2 distinct factors (*χ*^2^_1_=2811.5, *P*<.001). Again, we used CFA to construct a factor that comprised 7 items that we labeled “Trust Health Researchers” (*χ*^2^_21_=1725.4, *P*<.001, α=.87), and a factor that comprised 5 items that we labeled “Health Researchers Pursue a Greater Good” (*χ*^2^_6_=737.3, *P*<.001, α=.79). It is noteworthy that when we used the EFA to directly construct the 2 factors, the results were broadly the same.

Although the values we presented for website trust and website skepticism may seem low, a low correlation does not mean the items are poor. Indeed, it is not unusual for a scale with a respectable alpha (say .70 or higher) to have modest correlations among the scale items. The Cronbach α, the most common measure of internal consistency, is a function of the average interitem correlation and the number of items. Our measures of website trust and website skepticism had 2 items each, and the low correlation among the items for the 2 scales is neither surprising nor a cause for great concern. Moreover, the factor loadings for the items comprising each of these measures were high (all >.80), indicating a commonality among the items and giving us confidence in our measures.

### Participants Endorsement of Privacy Concerns

Table 2 presents the mean responses to our various measures of privacy concerns. The mean response to the measure of trust scale was 2.57 (see the first column of numbers), which is significantly below the scale midpoint of 3 (*t*_360_=11.78, *P*<.001), suggesting that nonresponders were on average distrustful of health websites (disagreement with the statement, “I trust that most health websites have systems in place to protect my privacy”). Conversely, the mean response to the measure of skepticism was 2.46, which is also significantly below the scale midpoint of 3, suggesting that nonresponders were not overly skeptical of the information on websites in general (*t*_361_=15.42, *P*<.001). For the views of health researchers, the grand mean for both measures was significantly greater than the scale midpoint of 3. Nonresponders reported that they trusted university health researchers (*t*_351_=20.07, *P*<.001), and believed university health researchers were pursuing the greater good (ie, striving to improve people’s lives; *t*_356_=30.68, *P*<.001).

**Table 2:** Responses to the trust measures as a function of group. Participants completed all items using a 5-step scale (1=strongly disagree; 5=strongly agree). Racial or ethnic minority refers to participants who self-identified as coming from a racial or ethnic minority group (self-identified as ethnically Hispanic or as non-White in race) or the majority group (self-identified as non-Hispanic in ethnicity and White in race). *B* represents the unstandardized regression coefficient.

Measure of trust	Grand mean			Racial or ethnic minority status						*B*			*t*	*P* values		
	Mean		SD	Majority		Racial or ethnic minority										
				Mean	SD		Mean	SD								
Interest in ovarian cancer information	3.48	0.68		3.42	0.68		3.66	0.65	–.238			–2.92			.004	
Benefit of visiting health website	3.50	0.72		3.39	0.72		3.78	0.64	–.396			–4.63			<.001	
Website trust	2.57	0.69		2.47	0.66		2.84	0.70	–.365			–4.56			<.001	
Website skepticism	2.46	0.66		2.53	0.64		2.28	0.70	.253			3.24			.002	
Trust health researcher	3.60	0.56		3.56	0.53		3.70	0.62	–.139			–2.04			.05	
Health researchers pursue greater good	3.88	0.54		3.84	0.53		3.97	0.55	–.131			–2.02			.05	

### Reasons for Not Visiting the YFC Website

Regarding our first research question, analysis showed that racial or ethnic minority status was significantly associated with privacy concerns regarding health websites. Compared with nonresponders who self-identified as nonminority, nonresponders who self-identified as part of a racial or ethnic minority group reported greater interest in ovarian cancer information, saw greater benefits to visiting the website, were more trustful and less skeptical about health websites, trusted in health researchers more, and were more likely to agree that university health researchers were pursuing a greater good (see Table 2).

### Association of Ovarian Cancer Relevance and Privacy Concerns With Recall of Prior Correspondence, Time Since Diagnosis, Age, and Race

To answer our second research question, we tested associations of the 6 factors with recall, time since diagnosis, age, and minority status. Of the nonresponders with confirmation of a correct mailing address, 196 responded “yes” they recalled the invitation, 99 responded “no,” and 65 responded “not sure.” It is noteworthy that most of these nonresponders were still at the same address where we mailed the original survey. We initially examined whether age, minority status (racial or ethnic minority, majority), or perceived benefits and risks differed between the 3 recall groups. Analyses revealed an effect only for age (*F*_2, 358_=5.89, *P*=.003). Nonresponders who recalled receiving the packet of materials were younger (mean 67.78, SD 12.18 years) than were those who reported they did not recall (mean 71.46, SD 11.79 years; *P*=.02), and younger than nonresponders who reported they were unsure they received the packet (mean 72.65, SD 9.62 years; *P*=.004). The latter 2 groups did not differ in age. Given these findings, we collapsed across these 2 groups in all other analyses, resulting in 196 nonresponders classified as reporting they received the packet of materials and 165 reporting they did not or were not sure.

Table 3 presents the correlation among all variables examined in our study, including our dichotomous recall measure. Because the demographic variables (age, time since diagnosis, and racial or ethnic minority status) were the 3 features linked to participating versus not participating in the parent study, our primary interest with the correlations was in the association between these 3 demographic measures and our 6 measures. As evident in Table 3, only racial or ethnic minority status correlated with measures of concern. The pattern of correlations is consistent with the results from the regression analyses we described.

**Table 3:** Correlation and corresponding probability values among predictors.

			Recall		Age		Time since diagnosis		Racial or ethnic minority Status		Interest in ovarian cancer information		Benefit of visiting health websites		Website trust		Website skepticism		Trust in health researchers
**Age**																			
	*r*	–0.17		—^a^		—		—		—		—		—		—		—	
	*P* value	<.001		—		—		—		—		—		—		—		—	
**Time since diagnosis**																			
	*r*	0.00		0.07		—		—		—		—		—		—		—	
	*P* value	.93		.20		—		—		—		—		—		—		—	
**Racial or ethnic minority status**																			
	*r*	0.02		0.07		0.09		—		—		—		—		—		—	
	*P* value	.76		.18		.09		—		—		—		—		—		—	
**Interest in ovarian cancer information**																			
	*r*	0.05		–0.07		0.00		0.16		—		—		—		—		—	
	*P* value	.31		.20		.98		.003		—		—		—		—		—	
**Benefit of visiting health websites**																			
	*r*	–0.02		0.04		0.00		–0.24		0.55		—		—		—		—	
	*P* value	.69		.49		≥.99		<.001		<.001		—		—		—		—	
**Website trust**																			
	*r*	0.00		–0.04		–0.06		–0.24		0.07		0.19		—		—		—	
	*P* value	.95		.47		.29		<.001		.18		<.001		—		—		—	
**Website skepticism**																			
	*r*	0.00		–0.04		0.05		0.17		–0.31		–0.53		–0.20		—		—	
	*P* value	.96		.41		.36		<.001		<.001		<.001		<.001		—		—	
**Trust in health researchers**																			
	*r*	0.06		0.07		–0.03		–0.11		0.27		*0.34*		0.07		–0.34		—	
	*P* value	.25		.18		.52		.05		<.001		<.001		.22		<.001		—	
**Health researchers pursue greater good**																			
	*r*	0.03		–0.02		0.02		–0.11		0.43		0.41		–0.01		–0.36		0.66	
	*P* value	.52		.76		.76		.05		<.001		<.001		.91		<.001		<.001	

^a^Not applicable.

### Profiles of Nonresponders to Encourage Use of Similar Websites

To address our third research question, we conducted a logistic regression analysis to explore how well we could classify nonresponders in the 2 recall groups. The analysis revealed that age was the only significant predictor of recall (Table 4), a finding consistent with the correlations reported in Table 3. When we included all predictors (age, racial or ethnic minority status, and the 6 privacy concerns) in the model, the model correctly classified 58.8% of nonresponders in the “yes” and “no or unsure” groups and incorrectly classified 41.2% of nonresponders. This rate of successful classification was not appreciably better than a logistic regression analysis that included only age as a predictor (57.7% survivors classified correctly, 42.3% of nonresponders classified incorrectly).

Racial or ethnic minority status distinguishes participants who self-identified as coming from a racial or ethnic minority group (ethnically Hispanic or non-White in race) and was coded as 0 versus participants who self-identify as White and non-Hispanic and was coded as 1. *B* represents the unstandardized regression coefficient.

**Table 4:** Results from logistic regression of survivor’s recall of receiving study information. *B* represents the unstandardized regression coefficient.

Predictor	*B*	SE	Chi-square (*df*)	*P* values
Age	.035	.01	10.9 (305)	.001
Racial or ethnic minority status	–.245	.28	0.8 (305)	.39
Interest in ovarian cancer information	–.301	.22	1.9 (305)	.16
Benefits of visiting health website	.296	.23	1.7 (305)	.19
Website trust	.014	.18	0.01 (305)	.94
Website skepticism	–.096	.22	0.2 (305)	.66
Trust in health researchers	–.437	.29	2.3 (305)	.13
Health researchers pursue greater good	.095	.31	0.1 (305)	.76

## Discussion

### Principal Findings

Our parent study revealed that far fewer (only 18%) survivors of ovarian cancer who were sent invitations to participate in our study visited the study website [3]. The invitation informed survivors that the website provided information about ongoing ovarian cancer risk for themselves and their at-risk relatives and free genetic counseling. With broadened internet access, website-centered health promotion approaches have proliferated. Yet researchers know little about people who do not engage. Our survey of a population-based sample of these nonresponders focused on the role of trust in visiting a health-related website. Researchers have noted that privacy concerns can inhibit the sharing of health information [8]. Given the sensitive nature of inherited cancer risk communication, the results of our preliminary investigation of ovarian cancer survivors [14], and the response of many of the survivors we attempted to recruit for our parent study, we posited that privacy concerns may be especially important.

We found that nonresponders were, on average, relatively distrustful of health websites as a platform. However, they were not especially skeptical of the information that would be provided on the website. Moreover, nonresponders generally reported that they trusted university health researchers and believed these researchers were pursuing the greater good. We found no demographic profile of nonresponders who were especially distrustful or skeptical. Applying the Extended Privacy Calculus framework to these results, we posit that the benefits of the information and services provided and the recognized good intentions of the researchers may have been insufficient to overcome ovarian cancer survivors’ distrust of a web platform.

It is worth remembering that 156 (45.5%) of the nonresponders in our sample reported that they did not receive or were unsure they received the mailed materials we sent encouraging them to visit our website. The GCR reconfirmed that the contact information used for the study invitations was accurate, suggesting the invitations were received. Although they did not visit an internet-based research website, these nonresponders all completed this current cross-sectional survey about their nonparticipation. At first blush, this finding suggests that interventions need to find ways to ensure that study invitations provide ample assurance of confidentiality. Yet, nonresponders’ recall of receiving the invitation was not associated with privacy concerns. Indeed, more than half of the nonresponders were basing their judgments regarding privacy without having reviewed anything about the website.

If trust in the website platform played a role in study nonparticipation, it raises an important question: what can interventions do to address audience distrust of websites, particularly when the website involves inherited risk and offers genetic services to close relatives? Another study used relationship-building with stakeholders to encourage website access. Shankar et al [22] suggest that website trust is a complex construct that comprises offline (eg, an organization’s reputation) and online (eg, emotional comfort with the site) dimensions of trust. They propose stakeholder-guided strategies for building trust. These strategies include (1) offline and online cues aimed to establish a consistent tone, (2) use of virtual-advisor technology, and (3) building a perception of a balance of power between the organization and the user to build web trust [22]. We worked with survivors and relatives who served as citizen scientists to identify endorsements that would enhance our website’s credibility and, as a result, used the logos of our school of public health and the GCR in all our contacts with survivors [14]. Indeed, survivors appeared to trust in these organizations, but their endorsement did not prompt them to participate.

To the extent that mistrust and concerns with privacy are driving participation rates, future web-based interventions for cancer survivors may consider partnering with organizations that have high credibility within the survivor community (eg, FORCE [Facing Our Risk of Cancer Empowered]) in a stepped process of relationship building before offering access to websites [22]. Such partnerships might enhance emotional comfort with websites and make relationship-building more sustainable. Engaging in a lead-in time frame and using serialized communications to promote website trustworthiness also warrants evaluation.

Contrary to our expectations, nonresponders who self-identified as belonging to a racial or ethnic minority group reported greater interest in ovarian cancer information, saw greater benefits to visiting the website, were more trustful and less skeptical about health websites, trusted in health researchers more, and were more likely to agree that university health researchers were pursuing a greater good. This effect held even when we controlled for age and time since diagnosis. Others have found similar results [23]. One of the advantages of websites is that survivors can engage with inherited risk information without directly involving health care providers. This less personal and more anonymous platform for information may promote trust among racial or ethnic minority survivors who have experienced interpersonal discrimination (such as not being referred for genetic counseling) from health care providers [24]. Importantly, our speculation that experiences of personal discrimination in in-person interactions within health care systems may underlie the greater trust in websites reported by the racial or ethnic minority members in our sample awaits confirmation in future research. And stepping back, few studies examine how racial or ethnic minority groups may differ in their responses to different types of research outreach approaches.

This study has several notable strengths, including a population-based sampling approach and a response rate, among nonresponders of more than 50%. However, our study had limitations. To keep the survey short and raise participation, we focused our measures on the conceptual domain of trust in websites and research. Consequently, we lacked information on other barriers that may have dissuaded survivors from participating. For example, we heard anecdotally that older participants were less confident that they could use the website effectively and safely. Our short survey also precluded delving into reasons for distrust and other psychosocial factors that researchers have shown influence participation in cancer research, such as levels of cancer worry. Further research through qualitative methods could explore these themes in depth. Although skepticism about websites and concerns with privacy that emerged from our parent study were the impetus for this project, we cannot conclude from this study that skepticism and privacy concerns were responsible for the low participation rate in our parent study. It is also noteworthy that we have no information on whether some survivors in our parent study were disinterested in visiting the website because they had already received genetic counseling elsewhere. And we lack data on disease severity, precluding our ability to examine its effect on nonresponding.

However, our sample size enabled us to explore patterns of nonparticipation among racial or ethnic minority groups that appear to challenge notions that people from racial or ethnic minority groups—especially in the context of genetics—do not participate due to distrust. These findings suggest a more complicated picture of nonparticipation and the potential that fostering trust in web platforms warrants intervention focus.

Finally, we were unable to sample all nonresponders in our study. Many nonresponders did not respond to our survey, and we do not know why. However, we noted early on that several survivors voiced strong concerns about privacy to us or to the Georgia State Cancer Registry in response to our contact during the parent study. Although having a larger sample of nonresponders in our study may have changed the results, we can only imagine that the effects we observed for privacy would have been even stronger had they participated.

### Conclusions

Although electronic platforms and health research websites appear to be the future for public health interventions, studies suggest that people may be disinclined to visit such sites due to privacy concerns. The nonresponders in the present study—particularly the White nonresponders—were skeptical of website platforms regardless of whether they recalled receiving a website invitation or not. Social marketing approaches geared toward building trust in web platforms by building a relationship with an information consumer and in collaboration with trusted organizations warrant further investigation. These strategies may optimize uptake and engagement of health-related websites to improve health and well-being.

## Appendix

Multimedia Appendix 1 Additional infographics.

## Abbreviations

CFA: confirmatory factor analysis
EFA: exploratory factor analysis
FORCE: Facing Our Risk of Cancer Empowered
GCR: Georgia Cancer Registry
YFC: Your Family Connects
