# Supplementary material for: Trust and Privacy Concerns Among Cancer Survivors Who Did Not Visit a Research Website Offering Free Genetic Counseling Services for Families: Survey Study
Source: J Med Internet Res. 2025 May 6;27:e64228. doi: 10.2196/64228 (PMC12093066; doi:10.2196/64228)
Supplement: Multimedia Appendix 1 [file jmir_v27i1e64228_app1.pdf]

EMORY | ROLLINS SCHOOL OF PUBLIC HEALTH

Ms. Hermione Farthingale
303 Bond Street
London, GA 30000

Resources for your family's health

CONFIDENTIAL

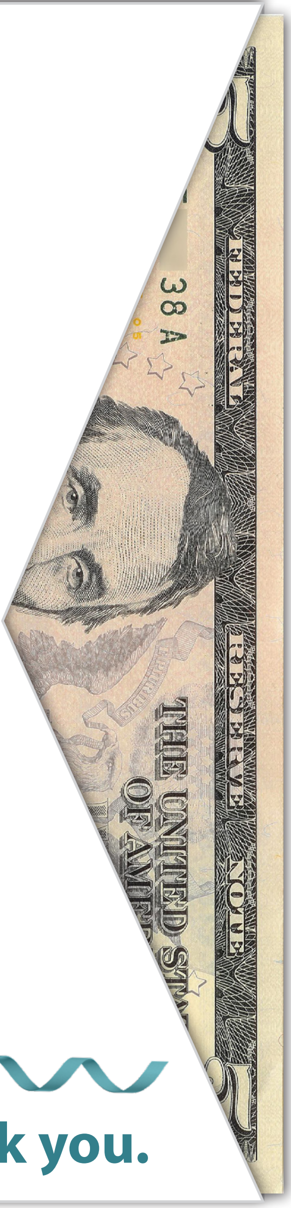

thank you.

GEORGIA CANCER REGISTRY

Connecting Families to Overcome Ovarian Cancer

EMORY | ROLLINS SCHOOL OF PUBLIC HEALTH

Dear <Registry Person First Name> <Registry Last Name>,

My name is Colleen McBride and I lead a team of cancer researchers from Emory University with funding from the National Cancer Institute (NCI – U01CA240581). Our research project, "Connecting Families to Overcome Ovarian Cancer", is reaching out to families affected by ovarian cancer to encourage discussions about their shared risk for cancer. We are collaborating with the Georgia Cancer Registry to recruit individuals for this study.

Every cancer diagnosed in Georgia is required by law to be reported and is entered into a statewide registry. This registry was designed to track the cancer burden among Georgia residents and to support studies about cancer to improve public health. The Georgia Cancer Registry has strict confidentiality and data use policies for the cancer surveillance data it is entrusted to collect.

The registry is reaching out to you now to invite you to consider participation in this study. No information that identifies you personally will be released to the research team without your authorization. If you have never had ovarian cancer and we have contacted you by mistake, please notify us of by contacting Dr. Kevin Ward at (404) 964-5796. We apologize for the inconvenience.

Our study is evaluating ways to improve ovarian cancer survivors' and their close relatives' understanding of their risk for ovarian and other forms of cancer and offering free genetic counseling to those who want these services.

This mailing includes several things:

- This cover letter, which describes what we offer you and what we ask in return.
- An information sheet that provides specific details about the study.
- A thank you gift of $5 for taking time to read this letter and consider being part of our study.

WHAT WE OFFER

We have launched a website with funding from the NCI. We developed the website with the help of a team of ovarian cancer survivors and relatives. The website provides several important resources for you and your close relatives:

- **Information about Ovarian Cancer** – Survivors tell us that it can be hard to find information about ovarian cancer. We try not to repeat information you can find at other websites. We provide links to trusted websites in the United States about ovarian cancer.
- **Information about Who in Your Family is at Increased Risk** – Close female relatives of women who have had ovarian cancer may be at risk. And close male relatives may be at risk for other types of cancer. The website can help you identify which close relatives are at risk for ovarian or other types of cancer.
- **Guidance for Talking with Close Relatives about Their Cancer Risk** – Talking with close relatives about cancer can be hard. The webpage offers tips to ease into these talks.

yourfamilyconnects.org

- **Free Genetic Counseling** –We offer free genetic counseling for you and your close relatives. The counseling can help you decide if genetic testing would be useful for your family. The free genetic counseling is available for a limited time with funding from the NCI.

WHAT WE ASK OF YOU

We included $5 in this mailing. It's not much, but is a small thank you for two requests.

- **First, Keeping Reading** – We ask that you finish reading this letter and the other material in this mailing.
- **Second, Visit the Website** – We developed the website for you and your close relatives. It may seem that the cancer community overlooks ovarian cancer, particularly compared with breast cancer. Through this project, the NCI has directed federal funds to provide resources to ovarian cancer survivors and their close relatives.

WHY SHOULD YOU VISIT THE WEBSITE?

You are a survivor! A team of survivors of ovarian cancer and close relatives have helped us with this study. They tell us that their cancer was, and remains, a big part of their life. You also may find that your cancer experience now defines you and how you think about your life, your family, and your world.

- **You can use your ovarian cancer experience to make a difference** – You are in a unique position to help others—especially your close relatives—learn about their risk for ovarian cancer. What we learn from your participation in this study will help other women who are at risk for ovarian cancer.
- **The website can help you help your family** – The website offers guidance on how you can start a conversation, how to have the talk sensitively, and the key points to make. On the website, we provide an example letter you can edit and send to close relatives to invite them to the website. Or, if you prefer, you can give us permission to send a letter to the close relatives you name. We will then send them a letter that invites them to the website where they can schedule free genetic counseling.

THANK YOU FOR READING THIS FAR!

We know the $5 in this mailing is not what got you this far. Like all of us, you want to do what you can to protect your family and keep them healthy. We are doing this research because we want to make a difference in the lives of families affected by ovarian cancer. To learn more about what you can do to help your close relatives who may be at risk, please go to our website. Please log-in at **yourfamilyconnects.org** and use the below activation code to start your study participation.

Sincerely,

Colleen M. McBride, PhD
Study Leader
Connecting Families to Overcome Ovarian Cancer

Kevin C. Ward, PhD
Georgia Cancer Registry
(404)964-5796
kward@emory.edu

If you have questions about this study, please call our Georgia Cancer Registry Study Coordinator, Mackenzie Crawford, at (404)-727-2135, or email the Study Leader, Colleen McBride at colleen.marie.mcbride@emory.edu. You can also contact the Georgia Cancer Registry toll free at 1-855-458-8788.

yourfamilyconnects.org

GEORGIA CANCER REGISTRY

Connecting Families to Overcome Ovarian Cancer

EMORY | ROLLINS SCHOOL OF PUBLIC HEALTH

yourfamilyconnects.org

your code is:

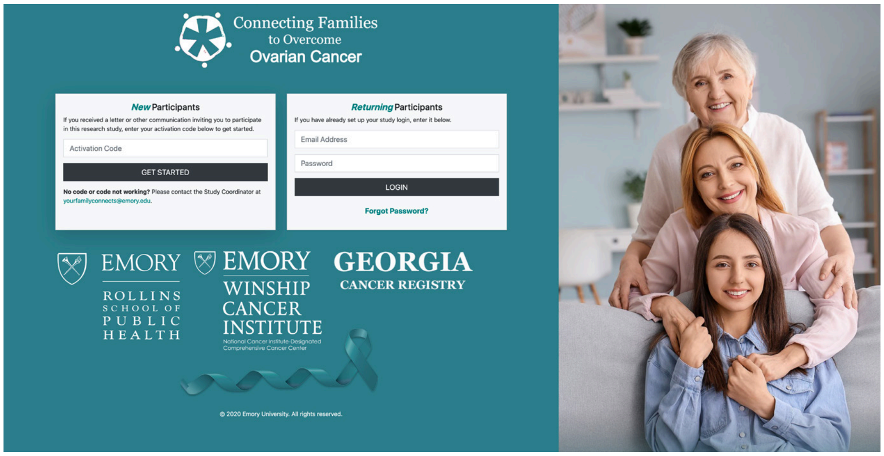

When you visit the weblink, you will see the website welcome page. Your activation code is above. You can then create a password to match your email address for future visits.

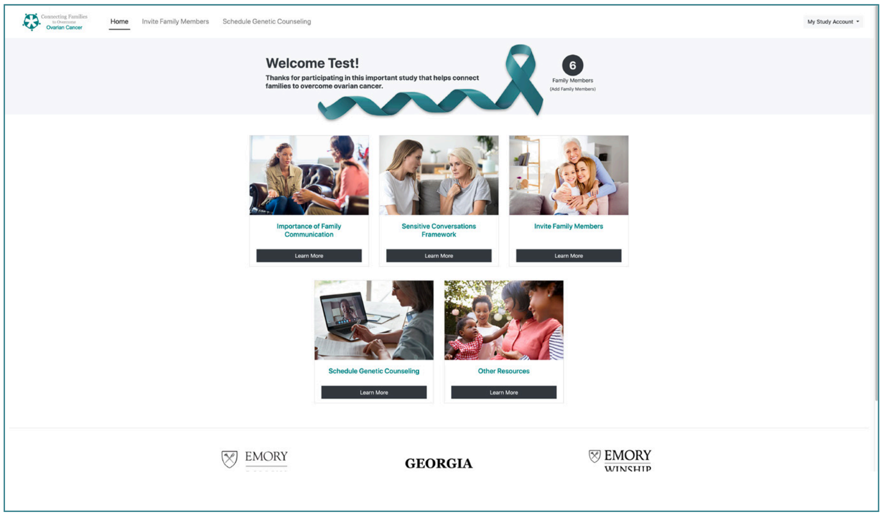

Once you enter the website, you will be guided through several tasks and then see this dashboard with links to resources for you and your family.
